# Potentially Toxic Elements in Terrestrial Mosses in the Vicinity of a Stibnite Mine in Pinal de Amoles, Mexico

**DOI:** 10.3390/plants14172657

**Published:** 2025-08-26

**Authors:** Samuel Tejeda, Graciela Zarazúa, Emma A. Juárez-Acosta, Carlos E. Barrera-Díaz, Luis R. León, Pedro Avila-Pérez, Carmen Zepeda-Gómez

**Affiliations:** 1 Instituto Nacional de Investigaciones Nucleares, Departamento de Estudios del Ambiente, Ocoyoacac 52750, Mexico; graciela.zarazua@inin.gob.mx; 2 Facultad de Ciencias, Universidad Autónoma del Estado de Mexico, Carretera Km 15.5, Piedras Blancas, Toluca 50200, Mexico; ejuareza001@alumno.uaemex.mx (E.A.J.-A.); zepedac@uaemex.mx (C.Z.-G.); 3 Centro Conjunto de Investigación en Química Sustentable UAEM—UNAM, Carretera Toluca-Atlacomulco, km 14.5, Unidad El Rosedal, Toluca 50200, Mexico; cebarrerad@uaemex.mx (C.E.B.-D.); pavilap@uaemex.mx (P.A.-P.); 4 Campus Pinal de Amoles, Universidad Autónoma del Estado de Querétaro, Fracción 3 del Predio “El Potrero”, Municipio Pinal de Amoles, Querétaro 76300, Mexico; luis.leon@uaq.mx

**Keywords:** potentially toxic elements, moss, soils, stibnite mine, enrichment factor

## Abstract

Mining waste often contains elevated concentrations of V, Cr, As, Sb, and Pb. Stibnite mining, during extraction and processing, generates waste that is deposited directly onto soil without vegetation cover, potentially leading to environmental pollution. This study assessed concentrations of potentially toxic elements (PTEs) in the rhizoids and stems-phyllidia of terrestrial mosses near antimony mines and used these mosses as biomonitors of soil contamination. Results obtained via energy-dispersive X-ray fluorescence spectrometry showed the highest concentrations of As, Sb, and Pb in mosses growing on mining rubble, reflecting elevated contaminant levels in the study area. Concentrations of As, Sb, and Pb differed significantly between mosses from mine rubble sites and those from forest and meander sites. Both rhizoids and stems/phyllidia of mosses from rubble sites showed high enrichment factors (EFs) for As, Sb, and Pb. Notably, PTEs concentrations in mosses from the forest area were lower than values reported for other regions, whereas concentrations in the mine rubble area exceeded those reported for other mining-polluted regions worldwide.

## 1. Introduction

The rapid growth of urban and industrial development has led to an increased presence of potentially toxic elements (PTEs) in the environment [1]. This increase is attributed to the indiscriminate discharge of PTEs from anthropogenic activities such as electroplating, painting, tanning, textile and dye production, paper manufacturing, and mining. These activities often release PTEs at concentrations exceeding permissible limits, resulting in the formation of soluble salts that pollute the atmosphere, water, and soil. Consequently, monitoring PTEs has become essential for ensuring environmental safety [2,3,4,5].

Since the 1960s, mosses have been employed as biomonitors of PTE pollution [6] due to their ability to accumulate substantial amounts of these elements in their tissues. Mosses serve as effective biomonitors for assessing environmental conditions such as air pollution [7,8,9,10,11,12], water pollution [13,14], and soil pollution [15,16,17], owing to their diverse habitats, simple structure, and high colonization rates. Their physiological traits, including the direct uptake of nutrients from soil surfaces, water, and atmospheric deposition—facilitated by passive transport due to the absence of true roots and a vascular system—further enhance their utility [8,11]. Additionally, their high cell wall permeability to ion transport and a volume-to-surface-area ratio five to ten times greater than that of vascular plants contribute to their effectiveness [12].

Atmospheric transport of mineral particles results in their deposition on moss tissues [18]. Mosses acquire elements from substrates such as soil, rocks, or tree trunks via rhizoids or epidermal tissues, which often show elevated concentrations of toxic elements. The filamentous structures of terrestrial mosses, known as rhizoids, serve solely to anchor the plant to the substrate. Due to this non-selective absorption mechanism, mosses function as excellent biomonitors of environmental quality by accumulating heavy metals and other contaminants from both atmospheric deposition and the surface substrate. The accumulation and storage of PTEs within the phyllidia or stems are attributed to direct uptake through the phyllid surface [19].

Estimates suggest that Mexico’s moss flora comprises around 1000 species, representing nearly 8% of the global moss flora [20]. Within the state of Queretaro, the moss flora includes 225 species, constituting 22.88% of Mexico’s total moss diversity [21]. Terrestrial mosses in the Sierra Gorda develop in humid temperate forest soils, predominantly on red chromic Luvisols, which are highly susceptible to erosion, have low organic matter content, a slightly acidic pH, and stony surfaces [22]. Luvisols are often associated with secondary soil types, including Lithosols, Rendzinas, Phaeozems, and, in some areas, calcareous Cambisols. These soil associations typically exhibit a medium to fine texture, dark grayish-brown color, clay crumb structure, and a depth of less than 50 cm [23,24].

Since 1990, several European countries have conducted studies on the trace elements accumulated in mosses to evaluate air quality at a transnational level, reporting 13 elements (As, Cd, Cr, Cu, Fe, Hg, Ni, Pb, V, Zn, Al, Sb, and N). Most studies used species such as *Pleurozium schreberi*, *Hypnum cupressiforme*, *Hylocomium splendens*, and *Pseudoscleropodium purum* [12,25,26,27]. Research using mosses as biomonitors is expanding in Mexico. In the State of Mexico, several studies have assessed air quality using mosses. Several works [8,10,11,28,29,30] involved collecting and/or deploying mosses in urban areas, leading to the detection of elevated concentrations of potentially toxic elements (PTEs), including Cd, Cr, Pb, and Zn. Mosses are also valuable for assessing soil pollution in mining areas [9,16,31]. The enrichment factor (EF) is commonly applied to determine the contribution of PTEs from the soil. This factor establishes a ratio between the concentration of PTEs in mosses and that in the underlying soil [15].

Some large-scale studies use different moss species because the restricted distribution of many species sometimes makes it impossible to base biomonitoring studies on a single species, making it necessary to use multiple species [31,32,33]. Additionally, both terrestrial and aquatic mosses have been found to be equally useful for heavy metal biomonitoring in highly polluted urban areas [30]. Furthermore, results from studies conducted in the same region can be compared over time since the greater the amount of particles deposited on the moss phyllid, the higher the concentration of PTEs. This supports that PTEs absorption by mosses occurs mainly through passive mechanisms dependent on the properties of the cell wall surface, with only a small percentage of the total concentration accumulating within the tissues, either in soluble form or bound to the internal plasma membrane [32,34].

**Figure 1:**
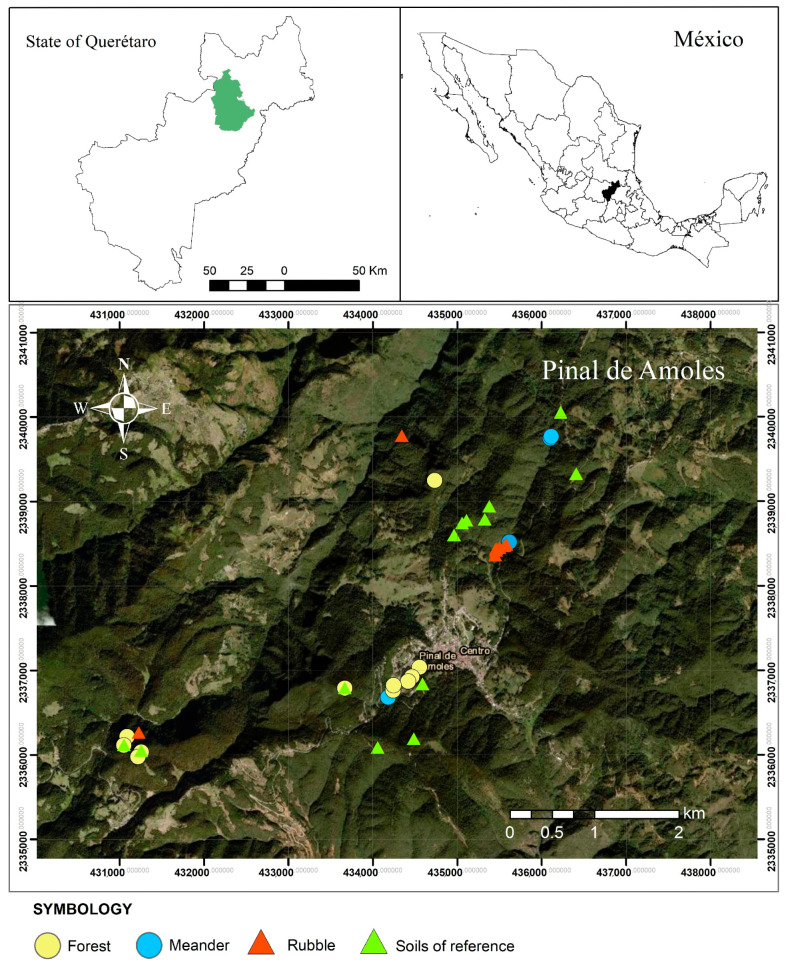
Location of sampling sites in the study area.

Pinal de Amoles, located in the Sierra Gorda of Queretaro (Figure 1), is characterized by complex Cretaceous sedimentary geology influenced by tectonic and magmatic events [35]. The region’s rich mineral deposits, especially hydrothermal mercury, silver, gold, and antimony—have historically driven mining operations [36,37]. In the Sierra Gorda region of Queretaro, stibnite ore is extracted from more than 40 mines in Pinal de Amoles, with approximately a dozen of these mines now abandoned. Terrestrial moss colonies were observed growing on three distinct substrates within the study area: rubble from two abandoned stibnite mines, surrounding pine forest soils, and sediments from the nearby Angel Stream.

Waste generated by the mining of stibnite (Sb_2_S_3_) and other associated minerals is a significant source of As and Pb pollution. These elements migrate into adjacent soils through surface runoff and infiltration into the soil profile, leading to anomalous enrichment of As and Pb in these soils and the mosses growing on them [38,39,40,41,42,43].

This study aims to comprehensively assess the long-term environmental impact of historical antimony mining activities on pristine natural soils in the under-researched alpine ecosystem of Pinal de Amoles, Queretaro, Mexico. Specifically, we determined the concentrations of Al, V, Cr, As, Sb, and Pb in five terrestrial moss species collected from forest, mine rubble, and meander sites near an abandoned stibnite mine. This research evaluates the biomonitoring potential of naturally growing terrestrial mosses for a comprehensive range of metals and metalloids in a forest environment, offering a novel, cost-effective, and ecologically relevant approach to assessing soil contamination caused by stibnite mine wastes.

## 2. Results and Discussion

### 2.1. Characteristics of the Moss Species Collected

Taxonomic identification of moss samples from the sampling sites revealed five distinct species. *Didymodon fallax* var. *reflexus* (Brid.) R.H. Zander, *Thuidium delicatulum* var. *delicatulum* (Hedw.) Schimp., and *Taxiphyllum deplanatum* (Bruch & Schimp. ex Sull.) M. Fleisch. were recorded in forest areas; *Archidium donnellii* (Austin) Lesquereux & James was found in rubble sites; and *Isopterygiopsis tenera* (Sw.) Mitt. was observed in meander areas. Table S3 in the Supplementary Material summarizes the morphological traits of the stems, phyllid, costa, and laminar cells for these five species identified within the study area. All taxonomic features were described according to standard taxonomic keys [44,45]. See Figure S2 in the Supplementary Material for additional illustrations.

### 2.2. PTEs Content in Reference Soils and Mosses

The median concentrations of potentially toxic elements (PTEs) in soil collected from the forest site were V (350 mg kg^−1^), Cr (78 mg kg^−1^), As (158 mg kg^−1^), Sb (42 mg kg^−1^), and Pb (127 mg kg^−1^). These reference values for V, Cr, and Sb are notably higher than those reported in Europe, Brazil, and Mexico [46,47,48], which document values of V = 60.4 mg kg^−1^, Cr = 60 mg kg^−1^, and Sb = 0.60 mg kg^−1^, aligning with similar findings in Brazil, Spain, and Mexico. Likewise, Pb and As concentrations were also higher than those reported for topsoil in Europe [46] and for soils polluted by tailings in northern Mexico [49], which showed mean values of 22.6 mg kg^−1^ and 7.03 mg kg^−1^ for Pb and As, respectively. In areas affected by mining, concentrations of As, Sb, and Pb in soils can be up to three orders of magnitude above the range of 30–100 mg kg^−1^, especially near mine dump sites [50]. These PTE reference values were subsequently used to calculate the enrichment factor in mosses within the area impacted by the Pinal de Amoles mine tailings. For more detailed information, see Table S5 in the Supplementary Material.

**Figure 2:**
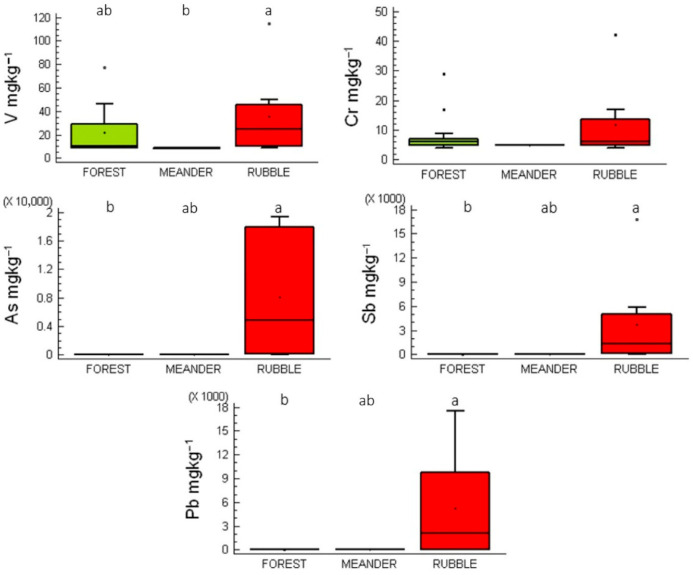
Box-and-whisker plots of V, Cr, As, Sb, and Pb concentrations in moss stems/phyllidia. Boxes sharing the same letter at the top indicate no statistically significant differences at *p* < 0.05.

PTEs concentrations in moss samples from the study sites (Figures S2 and S3, Supplementary Material) revealed that in mosses from rubble sites, median concentrations followed the trend As (4847 mg kg^−1^) > Pb (2082 mg kg^−1^) > Sb (1291 mg kg^−1^) > V (25 mg kg^−1^) > Cr (6 mg kg^−1^) (Figure 2). V concentrations were slightly higher in rubble-site mosses than in forest and meander sites; however, these differences were not statistically significant. In contrast, As, Sb, and Pb concentrations were markedly higher in rubble-site mosses than in those from forest and meander sites. Across all sampling locations, rhizoid tissues consistently showed significantly higher As, Sb, and Pb concentrations, approximately double those in stems/phyllidia, likely due to direct contact with substrate-bound PTEs.

Box-and-whisker plots illustrating V, Cr, As, Sb, and Pb concentrations across the three sampling site categories highlight notable variations in PTEs accumulation within stems/phyllidia, likely attributable to the high permeability of their cell wall surfaces [2]. Kruskal–Wallis and Dunn’s multiple comparison tests revealed statistically significant differences (*p* < 0.05) in V, As, Sb, and Pb concentrations among stems/phyllidia from forest, rubble mine, and meander sites. Conversely, Cr concentrations did not differ significantly (*p* > 0.05) between the three site types. Notably, As, Sb, and Pb concentrations in stems/phyllidia were significantly higher in rubble mine sites compared with forest and meander sites (Table S6, Supplementary Material).

**Figure 3:**
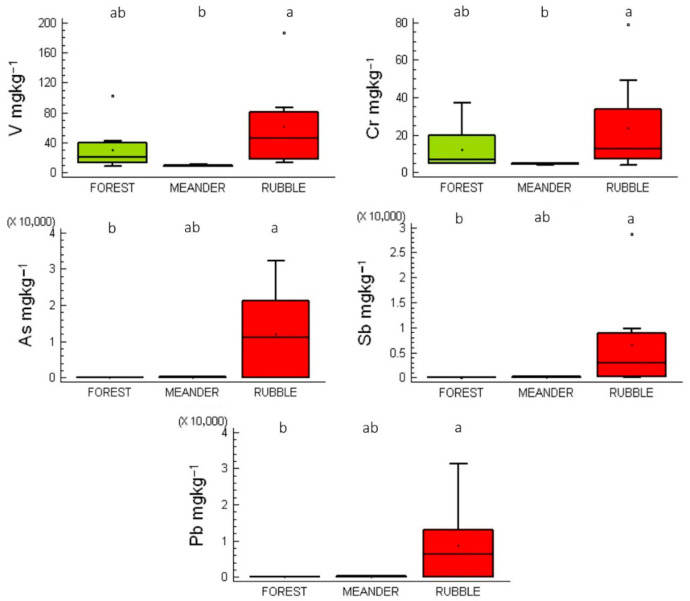
Box-and-whisker plots of V, Cr, As, Sb, and Pb concentrations in moss rhizoids. Boxes sharing the same letter at the top indicate no statistically significant differences at *p* < 0.05.

The concentrations of V in the rhizoids of mosses from forest, rubble, and meander sites were 21, 47, and 9 mg kg^−1^, respectively. Similarly, Cr concentrations in rhizoids from these sites were 7, 13, and 5 mg kg^−1^. However, these differences were not statistically significant (*p* > 0.05) (Figure 3).

In contrast, As, Sb, and Pb concentrations in rhizoids collected from rubble sites were significantly higher than those in mosses from forest and meander sites (*p* < 0.05). This elevated accumulation is primarily due to the direct contact of rhizoids with the substrate; when anchored to rubble, they are exposed to and incorporate higher concentrations of dissolved potentially toxic elements (PTEs) and contaminated soil particles.

**Figure 4:**
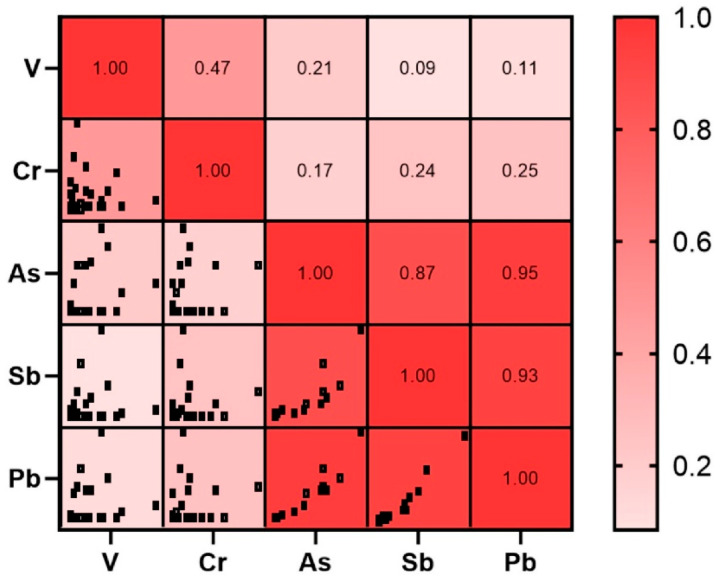
Graphics and values of Spearman correlation matrix of PTEs in mosses.

Figure 4 presents Spearman’s correlation results for PTEs analyzed in moss samples, including both rhizoids and stems/phyllidia. The data reveal strong positive correlations between As and Sb (r = 0.87, *p* < 0.01), Sb and Pb (r = 0.93, *p* < 0.01), and As and Pb (r = 0.95, *p* < 0.01), while a moderate positive correlation is observed between Cr and V (r = 0.47, *p* < 0.01). These strong correlations among the PTEs measured in mosses suggest that these elements may share the same or similar sources or are influenced by common factors, supporting their origin from mining activity [31].

Terrestrial mosses are widely recognized for their effectiveness in biomonitoring heavy metals in highly polluted areas. To achieve broad spatial coverage, multiple species are often required since elemental concentrations can vary between them. The observation that only one species was present in the mining rubble zones [30] may indicate superior tolerance to metal toxicity, reflecting the distinct sensitivities and enrichment capacities of different moss species [17]. Regardless of species, PTEs are primarily retained through adsorption, ion exchange, and both passive and active intracellular uptake. These processes are influenced by various factors, such as cell age. Therefore, green shoots were sampled in this study to maximize PTE accumulation, following established methodologies [12,32].

**Figure 5:**
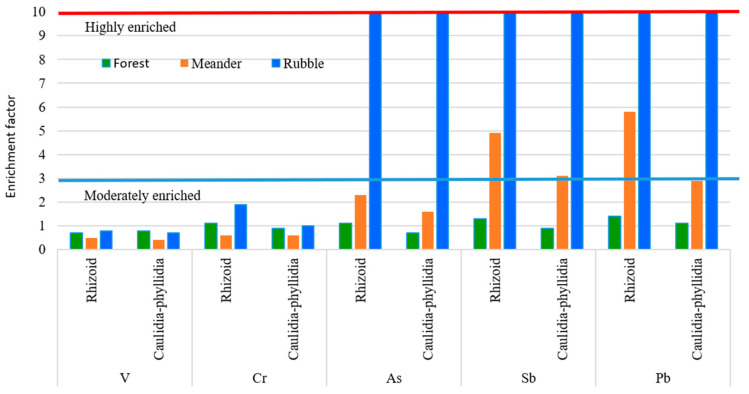
Enrichment factors of V, Cr, As, Sb, and Pb in rhizoids and stems/phyllidia of moss from forest (green), meander (red), and rubble (blue) sites.

Figure 5 illustrates the enrichment factors (EFs) of PTEs in mosses from the different sampling areas, as detailed in Table S6 of the Supplementary Material. No PTEs enrichment was observed in the rhizoids or stems/phyllidia of mosses from the forest area. Rhizoids and stems/phyllidia of mosses from the meander area exhibited moderate enrichment of Sb, with EF values of 3.1, 4.9, and 5.8. Rhizoids and stems/phyllidia of mosses from the mine rubble area showed no enrichment of V and Cr, with EF values of 0.8 and 1.9, respectively. However, these tissues exhibited high enrichment of As, Sb, and Pb, with EF values of 300 and 338 for As, 541 and 676 for Sb, and 287 and 362 for Pb, respectively. These high enrichment factors reflect the elevated concentrations of these elements found in the mine rubble area. It is important to note that the median concentrations of As, Sb, and Pb in forest site soils—158, 42, and 127 mg kg^−1^, respectively—which were used to calculate the enrichment factors, exceed the critical concentration thresholds for potential toxicity: 20 mg kg^−1^ for As, 5 mg kg^−1^ for Sb, and 100 mg kg^−1^ for Pb [50]. Therefore, PTEs’ presence at these elevated levels in forest soils near the mines poses a significant environmental risk, potentially threatening organisms associated with these soils. Furthermore, the median concentrations of As, Sb, and Pb in mosses from the mine rubble area exceed normal plant levels by approximately 692, 6455, and 104 times, respectively [51]. This demonstrates the high capacity of mosses to accumulate PTEs in areas heavily impacted by mine waste and simultaneously indicates a significant toxicity risk due to the elevated concentrations accumulated in their tissues.

Bryophytes have developed various mechanisms to combat the toxicity of intracellular PTEs, such as converting chemical species into less toxic forms and synthesizing antioxidants like phenolic compounds, β-carotene, and glutathione. These antioxidants reduce the incorporation of metals by sequestering and accumulating them in vacuoles [13,52]. Some studies have shown that mosses exposed to varying levels of environmental pollution can survive, although their vitality may decrease by more than 50% during exposure [53]. This decline indicates the adverse effects caused by high levels of environmental pollution, as also observed in this work. The bioavailability of PTEs—that is, the proportion of contaminants in soil and dust accessible for absorption by mosses, plants, and other organisms—represents the greatest risk to ecosystems. For PTEs to be bioavailable, they must be in a mobile form that organisms can freely take up and transport to target sites where they may pose risks [54].

The biological accumulation coefficient (BAC) is an index used to assess PTEs bioavailability in soil, calculated by dividing the PTEs concentration in plants by that in soil [55]. In this study, the enrichment factors (EFs) provide a reference framework for evaluating PTEs bioavailability in the area. The high EF values observed for As, Sb, and Pb suggest that these elements have high bioavailability in the mine waste area, are incorporated into moss tissues, and pose a significant potential risk of toxic and environmental effects to the ecosystem. Finally, metal-excluder plants are those that prevent the transport of absorbed heavy metals to their aboveground tissues, instead accumulating these metals in their roots [56]. Based on this definition, the moss species studied here appear to act as metal excluders by exhibiting the highest PTEs concentrations in their rhizoids and showing limited translocation of PTEs to their aerial parts.

Comparison of PTEs concentrations in the mine rubble mosses from this study with data from the literature (Table 1) shows that vanadium (V) and chromium (Cr) levels are lower than those reported for Gongga Mountain, China [31], and an active mine in Murgul, Turkey [57]. However, these concentrations are higher than values observed in the Colline Metallifere region, Italy [7,58], and Spain. Despite conservation efforts to protect natural resources, the forests of the Sierra Gorda remain vulnerable to the impacts of climate change and particulate pollution transported from anthropogenic activities at distant locations [59].

### 2.3. Analysis of Stems/Phyllidia Samples by SEM-EDS

**Figure 6:**
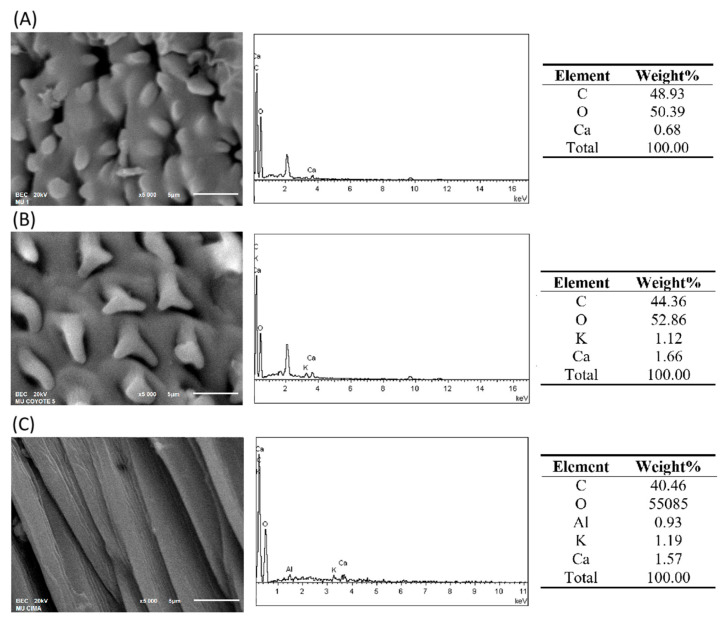
Micrographs, spectrum, and EDS results of moss phyllidia: (**A**) *D. fallax var reflexus*, (**B**) *T. delicatulum* var. *delicatulum*, and (**C**) *T. deplanatum*.

Figure 6A shows a micrograph of the adaxial surface of the phyllidia of *D. fallax* var. *reflexus*, where the distinctive papillae of this species collected in the forest are clearly visible. The EDS spectrum reveals characteristic peaks of C, O, and Ca at energies of 0.27, 0.52, and 3.69 keV, respectively. The analyzed epidermal area consists of 48.93% C, 50.39% O, and 0.68% Ca. Figure 6B presents a micrograph of the phyllidia epidermis of *T. delicatulum* var. *delicatulum*, showing the unipapillose cells with two beaks characteristic of the forest-collected species. The EDS spectrum indicates peaks of C, O, K, and Ca, with concentrations of 44.36%, 52.86%, 1.12%, and 1.66%, respectively. Figure 6C shows a micrograph of the epidermis of *T. deplanatum* collected from the meander site. The EDS spectrum of this area shows peaks of C, O, Al, K, and Ca, with concentrations of 40.46%, 55.85%, 0.93%, 1.19%, and 1.57%, respectively.

**Figure 7:**
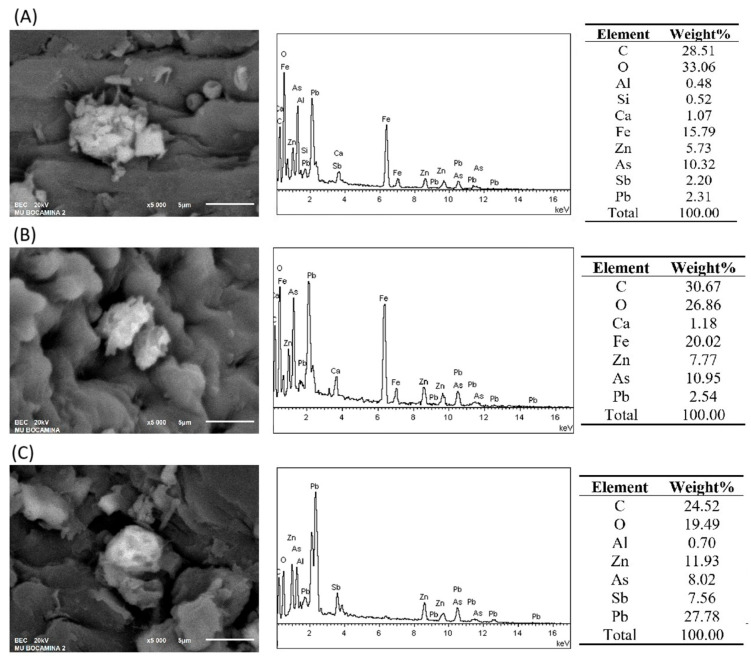
Micrograph, spectrum, and EDS result of the *A. donnellii* moss phyllidia from the mine rubble area. (**A**) Particle embedded within the internal tissue; (**B**) particles in the phyllidia, and (**C**) particle in the internal tissue.

Figure 7A presents a micrograph of *A. donnellii* collected from the mine rubble, showing a smooth epidermis and a particle embedded within the internal tissue. The corresponding EDS spectrum revealed peaks for C, O, Si, Ca, Fe, Zn, As, Sb, and Pb, with concentrations of 10.32% As, 2.20% Sb, and 2.31% Pb. Figure 7B shows another micrograph of a particle embedded within the tissues of the same species. Its EDS spectrum exhibited peaks for C, O, Ca, Fe, Zn, As, and Pb, with As at 10.95% and Pb at 2.54%. Figure 7C, also from *A. donnellii*, highlights a particle within the tissues. The EDS spectrum of this particle displayed characteristic peaks of C, O, Al, Zn, As, Sb, and Pb, with notably high concentrations of 8.02% As, 7.56% Sb, and 27.78% Pb. These results confirm the presence of As, Sb, and Pb within the internal moss tissues, consistent with the high concentrations detected by EDXRF in samples from the mine rubble area.

The presence of these particles in the phyllidia of *A. donnellii* indicates the transport of PTEs via dust particles lifted from the soil. This factor was controlled by washing the mosses prior to analysis. Since the greater the amount of particles deposited on the moss phyllid, the higher the PTEs concentrations, a portion of the total concentration also accumulates within the tissues. This occurs through the diffusion of metal cations via aqueous solutions from the soil to the mosses, contributing to overall accumulation [15,33]. The contribution of PTEs originating from the soil is also evaluated using the enrichment factor (EF), which compares relative concentrations in mosses to those in the substrate. In this study, mosses from rubble sites showed high enrichment, while mosses from meander and forest sites were not enriched, confirming that PTE absorption by mosses occurs partly via passive mechanisms [15].

The effectiveness of mosses as biomonitors for detecting PTEs in soils contaminated by stibnite mine tailings has been demonstrated in previous studies of abandoned mineral mines containing mercury, copper, iron, zinc, and lead. In the Sierra Gorda region, numerous abandoned stibnite and cinnabar mines remain unstudied using moss biomonitoring programs, presenting an opportunity to expand research to other areas affected by soil pollution from mercury, lead, arsenic, and other heavy metals.

## 3. Materials and Methods

### 3.1. Study Area

The Sierra Gorda region of Queretaro lies between 20°50′ and 21°45′ N latitude and 98°50′ and 100°10′ W longitude, covering an area of 383,587.5 hectares across the municipalities of Peñamiller, Pinal de Amoles, Jalpan de Serra, Landa de Matamoros (all within the State of Queretaro), and parts of Guanajuato (Figure 1). The region’s topography is characterized by convex slopes, steep gradients, canyons, and plains. Convex slopes, with gradients ranging from 12% to 70%, dominate the western area, while steep slopes with similar gradients prevail in the eastern and southern regions. Canyon slopes range from 40% to over 70%. The terrain is notably rugged, with elevations ranging from 300 to 3100 m above sea level, and an average altitude predominantly between 1300 and 2400 m. Notable elevations include Calentura and Pinguica hills, reaching 3060 and 3100 m above sea level, respectively, both located in the municipality of Pinal de Amoles (the State of Queretaro) [23].

The geological structures of the Sierra Gorda are characterized by two primary formation styles: thin-stratified limestone-clay and sandy-clay rocks as well as medium- to coarse-stratified calcareous rocks. The latter formation typically exhibits extensive anticlinal structures with abundant joints and fractures as well as small- to medium-scale normal faults [24].

The study area is located near the tailings of a stibnite mine in Pinal de Amoles [60]. Sampling sites were grouped into three categories—mining rubble, forest areas, and the meander of the Angel Stream—based on their similar characteristics to facilitate a clearer interpretation of the study area. These sites were mapped using ArcMap software (ArcGIS Desktop 10.8), as shown in Figure 1.

### 3.2. Sampling and Sample Preparation

At each of the 23 sampling sites, 5 to 10 moss samples were collected and combined into a composite sample containing approximately 1 L of fresh moss per site. Eight samples were taken from mining waste dumps, eleven from forest areas, and four from the riverbanks (meanders) of the Angel Stream (see Figure S1 and Table S1 in the Supplementary Material). Moss samples were collected using a plastic spatula that had been cleaned beforehand to ensure sample integrity. All samples were collected during the growing season, selecting actively growing shoots to maximize pollutant accumulation. Samples were then placed in pre-labeled polyethylene bags and transported to the laboratory in closed plastic containers for processing and analysis [32]. Additionally, thirteen uncontaminated soil samples were collected to serve as reference soils (Table S4). These samples originated from a coniferous forest located over 500 m from any antimony mine and at an altitude of 3000 m above sea level [61,62].

### 3.3. Taxonomic Identification

Moss species were identified using taxonomic guides [44,45], supplemented by taxonomic reviews and distribution data [63]. Macroscopic features examined included caulidium length, width, branching pattern, color, and presence of paraphyllia as well as phyllidia color, length, shape, aggregation, margin type, costa length, and apex morphology. These features were observed using a Cole Parmer EW-48920-20 stereoscopic microscope. Microscopic characteristics, such as basal, laminar, and marginal cell morphology along with capsule peristome structure, were examined with a Leica DM300 optical microscope. Photographic documentation of moss colonies was captured using a Sony A7RIII camera.

### 3.4. Sample Preparation

Moss samples were carefully washed with demineralized water to remove substrate residues from the rhizoids. Then, the green shoot parts (3–4 cm) were cut from the moss, rinsed with distilled water, and placed separately in aluminum trays. Both rhizoids and stems/phyllidia were air-dried at room temperature, ground using an agate ball mill, and sieved through a 100-mesh (150 μm) screen. For energy-dispersive X-ray fluorescence (EDXRF) analysis, 250 mg of each sample was pelletized into 13 mm discs using a die press at 5 tons of pressure. For scanning electron microscopy (SEM) analysis, portions of stems and phyllidia were mounted on carbon tape attached to aluminum stubs and sputter-coated with gold at 50 mTorr, 25 mA, for 60 s using a Denton sputtering apparatus.

### 3.5. Sample Analysis

Pellets from moss samples, soil reference samples, and reference materials were analyzed in triplicate using a Rigaku NEX GC energy-dispersive X-ray fluorescence (EDXRF) spectrometer (Rigaku Holdings Corporation, Tokyo, Japan) [28,64]. The spectrometer featured a Pd X-ray tube operating at 50 W and 50 kV, with four secondary targets arranged in Cartesian geometry. A Si-Li Flash^®^ Drift detector with a resolution better than 150 eV for Mn Kα was employed. Elements Al, V, Cr, As, Sb, and Pb were quantified using the fundamental parameter method with FPR-SQX FP^®^ software version 3.5. Quality control was maintained by analyzing four reference materials: IAEA-Soil5, IAEA-Soil7, NIST 1572, and NIST 1573 [10,65,66]. Although EDXRF is a multielement technique, this study focused only on five PTEs considered relevant from toxicological and environmental perspectives; therefore, other elements were not included in the analysis.

The internal tissue morphology of phyllidia from mosses collected in forest, meander, and mine waste sites was examined using a JEOL JSM 6610LV scanning electron microscope (SEM) (JEOL Ltd, Tokyo, Japan). Imaging employed backscattered electrons under high vacuum conditions at 20 kV with an 11 mm working distance. Chemical composition analyses of both the internal tissue and embedded particles were performed using an Oxford BSIC Si-Li energy-dispersive X-ray spectroscopy (EDS) detector Rev.1.2.10 (Oxford Instruments, Abingdon, Oxfordshire, UK). Spectra acquisition was performed with Oxford INCA x-act 2007 software (51-ADD0013) (Oxford Instruments, Abingdon, Oxfordshire, UK) at 5000× magnification and a 120 s integration time [67].

Precision and accuracy were evaluated by measuring elemental concentrations in the Standard Reference Material NIST 2710a “Montana I Soil” [68]. Measured values showed good agreement with certified reference concentrations, with recovery rates exceeding 94%. The coefficient of variation (CV) for all potentially toxic elements was below 7%. See Table S2 in the Supplementary Material.

### 3.6. Enrichment Factor

The enrichment factor (EF) is widely used in passive air quality monitoring when employing mosses as biomonitors of pollution. This factor enables the comparison of individual PTEs concentrations in mosses with those of a conservative element in reference soil. Aluminum is commonly selected as the conservative element because of its relatively uniform concentration in the Earth’s crust and soils and its minimal anthropogenic input at levels that could significantly alter its natural abundance [61,69]. EF estimation helps to distinguish between anthropogenic and natural sources of contaminants [70,71]. After determining the PTEs concentrations in moss and soil samples, the EF was calculated using the equation described in previous studies [71,72].

(1) $$EF=\frac{Mm/Xm}{Mo/Xo}$$

where EF is the enrichment factor.

Mm: Concentration of metal “x” in the moss sample.

Xm: Concentration of the conservative reference element (Al) in the moss sample.

Mo: Concentration of metal “x” in reference soil from the Pinal de Amoles forest.

Xo: Concentration of the reference conservative element (Al) in reference soil from the Pinal de Amoles forest.

The criteria for assessing the level of PTE enrichment levels in terrestrial mosses are the following: an enrichment factor ≤ 3 is considered not enriched (conservative), 4–9 is considered slightly to moderately enriched, and ≥10 is considered highly enriched [8,28,30].

### 3.7. Statistical Analysis

Data were analyzed using Statgraphics Plus 5.1. Because the data did not follow a normal distribution, results are presented as medians and standard deviations. Differences in PTEs concentrations among soil sites were assessed using the Kruskal–Wallis test, followed by Dunn’s multiple comparisons test, with significance set at *p* < 0.05. Spearman’s correlation analysis was performed to identify associations between PTEs concentrations; significant correlations suggest common or similar sources. Correlation coefficients, which indicate the strength of relationships between PTEs concentrations, were calculated using GraphPad Prism 10.4.6 and Statgraphics Plus 5.1, with significance defined as *p* < 0.01.

## 4. Conclusions

This study determined the concentrations of Al, V, Cr, As, Sb, and Pb in terrestrial mosses sampled near a stibnite mine in the Sierra Gorda. It also analyzed their taxonomic characteristics and explored potential pollution sources using enrichment factors.

Various moss species were identified across the three sampling sites. Since metal accumulation in mosses can be influenced by species-specific traits and substrate composition, these factors should be considered when using mosses as biomonitors for soil metal pollution.

The results confirmed that the substrate significantly influences PTEs accumulation in mosses. Specifically, As, Sb, and Pb concentrations in mosses from mine rubble were three orders of magnitude higher than those in forest mosses. In contrast, V, Cr, As, and Pb concentrations in forest mosses were within global ranges, whereas As and Pb concentrations in mine rubble exceeded those reported in other mining-impacted regions. Significant statistical differences were observed in As, Sb, and Pb concentrations between rhizoids and stems/phyllidia as well as among different sampling sites. These differences reflect greater PTEs accumulation in the mine rubble area compared to other sites and in rhizoids compared to other moss parts. Median concentrations of As, Sb, and Pb in forest site soils greatly exceed critical thresholds above which toxicity is considered likely, indicating a significant environmental risk and toxicity hazard for soil-associated organisms. The high enrichment factor values for As, Sb, and Pb suggest these PTEs have high bioavailability in the mining waste area and represent a significant potential toxic and environmental risk to the ecosystem. The moss species studied act as metal excluders or PTE excluders, accumulating the highest concentrations in their rhizoids without translocating PTEs to aerial parts.

This study demonstrates that terrestrial mosses in the Sierra Gorda are significantly enriched in As, Sb, and Pb, highlighting substantial environmental impact from mining activities. To better understand variations in enrichment factors within the biosphere reserve, expanding sampling to additional sites and increasing sample numbers in the Sierra Gorda is recommended.

## Figures and Tables

**Table 1:** Comparison of the PTE concentrations (mg kg^−1^) obtained in this study with those reported in similar moss studies worldwide.

PTE	Forest	Meander	Mine Rubble	Gongga Mountain, China ^1^	Murgul Mine, Turkey ^2^	Colline Metallifere Italy ^3^	Wanshan District China ^4^	Spain ^5^
V	10	2	25	---	70.9	1.4	---	---
Cr	6	1	6	8.3	43.4	5.1	---	2.6
As	10	42	4847	---	18.1	1.4	3.5	0.4
Sb	0.1	37	1291	---	---	---	---	---
Pb	10	50	2082	6.6	19.0	2.7	---	9.3

^1^ [31]; ^2^ [57]; ^3^ [58]; ^4^ [9]; ^5^ [7].

## Data Availability

All original data and materials presented in this study are included within the article and its Supplementary Materials. For further information or requests, please contact the corresponding author.

## Supplementary Materials

The following supporting information can be downloaded at https://www.mdpi.com/article/10.3390/plants14172657/s1. Figure S1: Sampling sites; Figure S2: Moss species; Figure S3: EDXRF analysis; Table S1: Sampling site mosses and UTM coordinates; Table S2: Comparison between measured and certified potential toxic elements (PTE) concentration for the NIST 2710a “Montana I Soil”; Table S3: Taxonomic characteristics of terrestrial moss species; Table S4: Concentration of PTE in forest soils in mgkg^−1^; Table S5: Results of PTE in terrestrial mosses obtained from forest, mine rubbles and meanders. Concentration in mgkg^−1^; Table S6: Enrichment Factor (EF) of PTE on the terrestrial mosses.

## Abbreviations

The following abbreviations are used in this manuscript:

PTE	Potentially toxic elements
