# Supplementary material for: Potentially Toxic Elements in Terrestrial Mosses in the Vicinity of a Stibnite Mine in Pinal de Amoles, Mexico
Source: Plants (Basel). 2025 Aug 26;14(17):2657. doi: 10.3390/plants14172657 (PMC12430368; doi:10.3390/plants14172657)
Supplement: Supplementary file 1 [file plants-14-02657-s001.zip › Table_S6.pdf]

Table S6. Enrichment Factor (EF) of PTE on the terrestrial mosses.

Metal/ metaloid	Structure	Forest	Meander	Rubble
		EF	***EF***	***EF***
V	Rhizoid	0.7	0.5	0.8
	caulidia-phyllidia	0.8	0.4	0.7
Cr	Rhizoid	1.1	0.6	1.9
	caulidia-phyllidia	0.9	0.6	1.0
As	Rhizoid	1.1	2.3	337.7
	caulidia-phyllidia	0.7	1.6	299.8
Sb	Rhizoid	1.3	4.9	676.2
	caulidia-phyllidia	0.9	3.1	540.7
Pb	Rhizoid	1.4	5.8	362.2
	caulidia-phyllidia	1.1	2.9	287.2
