# Supplementary material for: Potentially Toxic Elements in Terrestrial Mosses in the Vicinity of a Stibnite Mine in Pinal de Amoles, Mexico
Source: Plants (Basel). 2025 Aug 26;14(17):2657. doi: 10.3390/plants14172657 (PMC12430368; doi:10.3390/plants14172657)
Supplement: Supplementary file 1 [file plants-14-02657-s001.zip › Table_S1.pdf]

Table S1. Sampling site mosses and UTM coordinates.

SITE	TIPE SOIL	Latitude N	Longitude W
CARRISILLO	FOREST	*21.12585*	*-99.663621*
CARRISILLO	FOREST	*21.124914*	*-99.663975*
PUERTO EL TEJOCOTE	FOREST	*21.124183*	*-99.662*
MINA PUERTO EL TEJOCOTE	RUBBLE	*21.126265*	*-99.662291*
EL COYOTE ALAMBRADO	FOREST	*21.12366667*	*-99.6623611*
MINA ANTIMONIO	RUBBLE	*21.174251*	*-99.622533*
MINA ANGEL	RUBBLE	*21.145389*	*-99.62171*
MINA ANGEL	RUBBLE	*21.145888*	*-99.621599*
MINA ANGEL	RUBBLE	*21.146185*	*-99.621201*
ARROYO ANGEL	SEDIMENTO	*21.1468*	*-99.6201*
CAMPO SANTO VIEJO	FOREST	*21.133384*	*-99.63029*
CAMPO SANTO VIEJO	FOREST	*21.132348*	*-99.631164*
CAMPO SANTO VIEJO	FOREST	*21.131863*	*-99.631588*
TALLERE MECÁNICO	SEDIMENTO	*21.130144*	*-99.633869*
TALLERE MECÁNICO	FOREST	*21.130904*	*-99.633301*
TALLERE MECÁNICO	FOREST	*21.131432*	*-99.633235*
CERRO CRUZ DE PALO	FOREST	*21.1311*	*-99.6388*
CERRO LA GACHUPINA	FOREST	*21.153361*	*-99.628638*
CABAÑA CRUZ VERDE	SEDIMENTO	*21.15791*	*-99.615478*
CABAÑA CRUZ VERDE	SEDIMENTO	*21.158119*	*-99.615361*
MINA ANGEL	RUBBLE	*21.14608333*	*-99.62161111*
MINA ANGEL	RUBBLE	*21.14638889*	*-99.62063889*
MINA ANGEL	RUBBLE	*21.1465*	*-99.62041667*
