# Supplementary material for: Potentially Toxic Elements in Terrestrial Mosses in the Vicinity of a Stibnite Mine in Pinal de Amoles, Mexico
Source: Plants (Basel). 2025 Aug 26;14(17):2657. doi: 10.3390/plants14172657 (PMC12430368; doi:10.3390/plants14172657)
Supplement: Supplementary file 1 [file plants-14-02657-s001.zip › Table_S2.pdf]

Table S2. Comparison between measured and certified potential toxic elements (PTE) concentration for the NIST 2710a "*Montana I Soil*"

PTE	Certified values NIST 2710a Concentration mg/kg		SD* mg/kg	Experimental values Concentration mg/kg		SD* mg/kg	RE %	CV %
Al	5.95 %	±	0.05 %	5.77 %	±	0.08 %	97	- 3
V	82	±	9	80	±	7	97	- 3
Cr	23	±	6	22	±	3	96	- 4
As	1540	±	100	1532	±	65	99	- 1
Sb	52.5	±	1.6	50	±	2	95	- 5
Pb	5520	±	30	5500	±	42	99	- 1

*SD: Standard deviation: RE: Percentage of recoveries; CV: Coefficient of Variation.
