# Supplementary material for: Potentially Toxic Elements in Terrestrial Mosses in the Vicinity of a Stibnite Mine in Pinal de Amoles, Mexico
Source: Plants (Basel). 2025 Aug 26;14(17):2657. doi: 10.3390/plants14172657 (PMC12430368; doi:10.3390/plants14172657)
Supplement: Supplementary file 1 [file plants-14-02657-s001.zip › Table_S3.pdf]

Table S3. Taxonomic characteristics of terrestrial moss species.

Species	Caulidium	Phyllid	Costa	Laminar cells
Didymodon fallax var. reflexus	Irregularly branched.	Green to red-brown. 1.2 to 3 mm long. Ovalate-triangular to lanceolate, acute with the base scarcely ovate. Strongly recurved.	Shortly excurrent, covered ventrally by short-rectangular cells and dorsally by quadrate cells.	Upper cells thick-walled and papillae high.
Thuidium delicatulum var. delicatulum	Spreading or arched-ascending, 2-pinnate and partially frondose.	Erect-spreading when moist, from 0.3 to 1.4 mm long. Triangular-ovate, broad acumen.	Ending in the acumen well below the apex. Excurrent.	Irregularly oblong-hexagonal, coarsely unipapillose forked and curved.
Taxiphyllum deplanatum	Up to 2.5 cm long, irregularly branched.	Crowded and imbricate, soft, erect and smooth. 1-2.3 mm long. Ovate to lanceolate, acuminate. Margins plane and serrulate to serrate above the middle.	Lacking or very short and double.	Cells smooth. Alar cells quadrate to short rectangular in to several rows.
Archidium donnellii	2-8 mm high.	Lanceolate to narrowly ovate to lanceolate, acumminate. Margins narrowly recurved, entire serrulate above.	Strong, percurrent to short-exurrent.	Median and upper cells irregular with thick walls, basal cells quadrate to short rectangular.
Isoeptrygium tenerum	1-3 cm long. Simple or irregularly branched.	Flaccid, erect spreading and complanate. Smooth. Ovate to lanceolate, acuminate. Margins serrulate.	None or weak, short and doble .	Smooth, alar cells short-rectangular to quadrate in small groups.
