# Supplementary material for: Potentially Toxic Elements in Terrestrial Mosses in the Vicinity of a Stibnite Mine in Pinal de Amoles, Mexico
Source: Plants (Basel). 2025 Aug 26;14(17):2657. doi: 10.3390/plants14172657 (PMC12430368; doi:10.3390/plants14172657)
Supplement: Supplementary file 1 [file plants-14-02657-s001.zip › Table_S4.pdf]

Table S4. Concentration of PTE in forest soils in mgkg^{-1}.

ID	V	Cr	As	Sb	Pb
C01	572	82	158	42	152
C02	506	63	104	21	117
C03	456	108	147	32	95
C04	142	63	130	86	127
C05	350	141	192	135	222
C06	336	47	209	52	116
C07	361	83	440	59	211
C08	273	34	349	124	153
C09	402	57	142	34	80
C10	213	80	140	71	99
C11	435	113	190	40	227
C12	174	78	52	21	40
C13	284	53	249	31	154
Median	350	78	158	42	127
Mínimum	142	34	52	21	40
Máximum	572	141	440	135	227
Average	346	77	192	57	138
SD.	129	30	104	38	57
