# Supplementary material for: Potentially Toxic Elements in Terrestrial Mosses in the Vicinity of a Stibnite Mine in Pinal de Amoles, Mexico
Source: Plants (Basel). 2025 Aug 26;14(17):2657. doi: 10.3390/plants14172657 (PMC12430368; doi:10.3390/plants14172657)
Supplement: Supplementary file 1 [file plants-14-02657-s001.zip › Table_S5.pdf]

Table S5. Results of PTE in terrestrial mosses obtained from forest, mine rubbles and meanders. Concentration in $mgkg^{-1}$

PTE	Part	Forest		Mine rubble		Meander	
		Median	Standard deviation	Median	Standard deviation	Median	Standard deviation
V	Caulidia-phyllidia	10^{ab}	23	25^{a}	35	9^{b}	0.5
	Rhizoids	21^{ab}	27	47^{a}	59	9^{b}	1
Cr	Caulidia-phyllidia	6	8	6	13	5	0.5
	Rhizoids	7^{ab}	10	13^{a}	26	5^{b}	0.5
As	Caulidia-phyllidia	10^{b}	7	4847^{a}	8809	42^{ab}	29
	Rhizoids	26^{b}	15	11067^{a}	12077	89^{ab}	151
Sb	Caulidia-phyllidia	<LOD[b]	--	1291^{a}	5710	37^{ab}	21
	Rhizoids	21^{b}	3	2938^{a}	9670	70^{ab}	127
Pb	Caulidia-phyllidia	10^{b}	10	2082^{a}	6464	50^{ab}	30
	Rhizoids	20^{b}	11	6282^{a}	10671	89^{ab}	154

<LOD. Below limit of detection.

[a, b and c] Same letter means no statistical significant difference between sampling areas of caulidia-phyllidia and rhizoids ($P<0.05$).
