# Supplementary figures and images for: Potentially Toxic Elements in Terrestrial Mosses in the Vicinity of a Stibnite Mine in Pinal de Amoles, Mexico
Source: Plants (Basel). 2025 Aug 26;14(17):2657. doi: 10.3390/plants14172657 (PMC12430368; doi:10.3390/plants14172657)

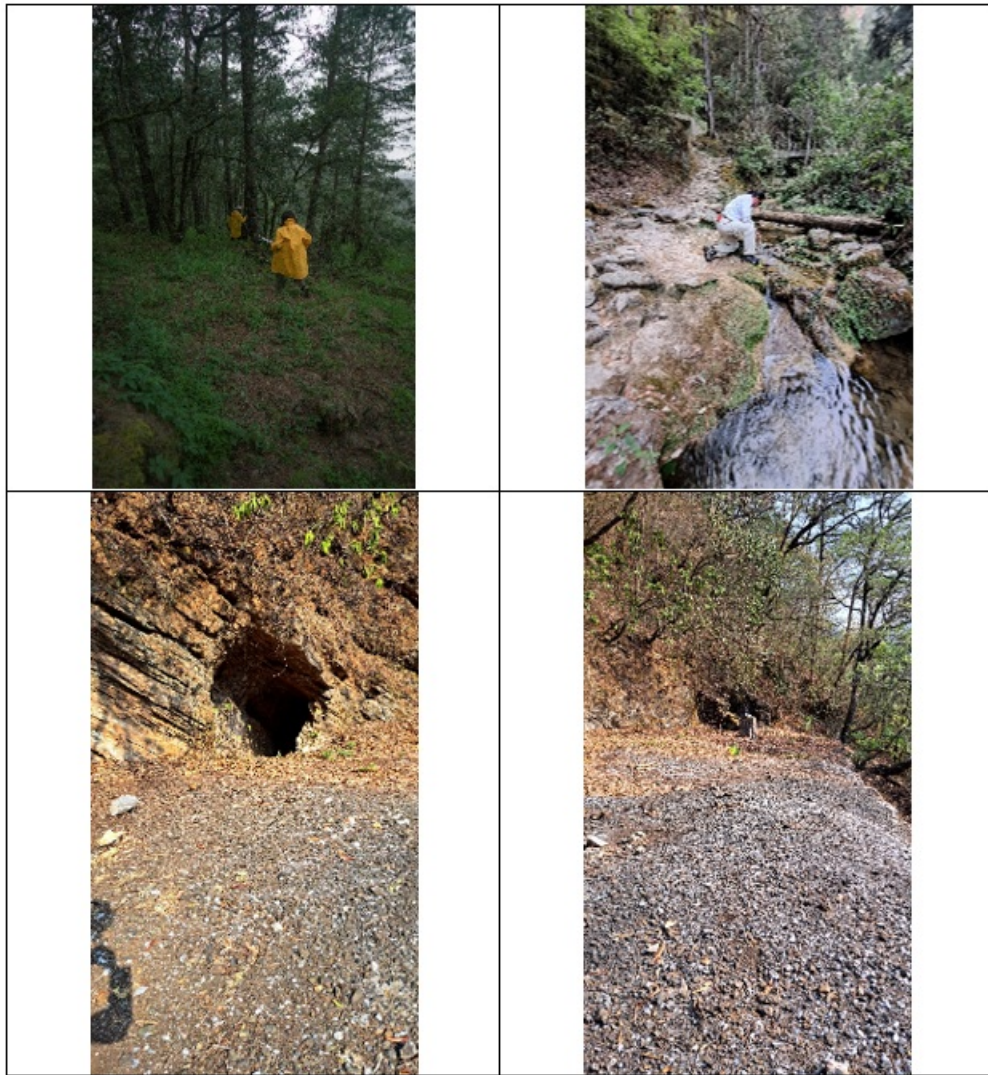

Figure S1: Sampling sites

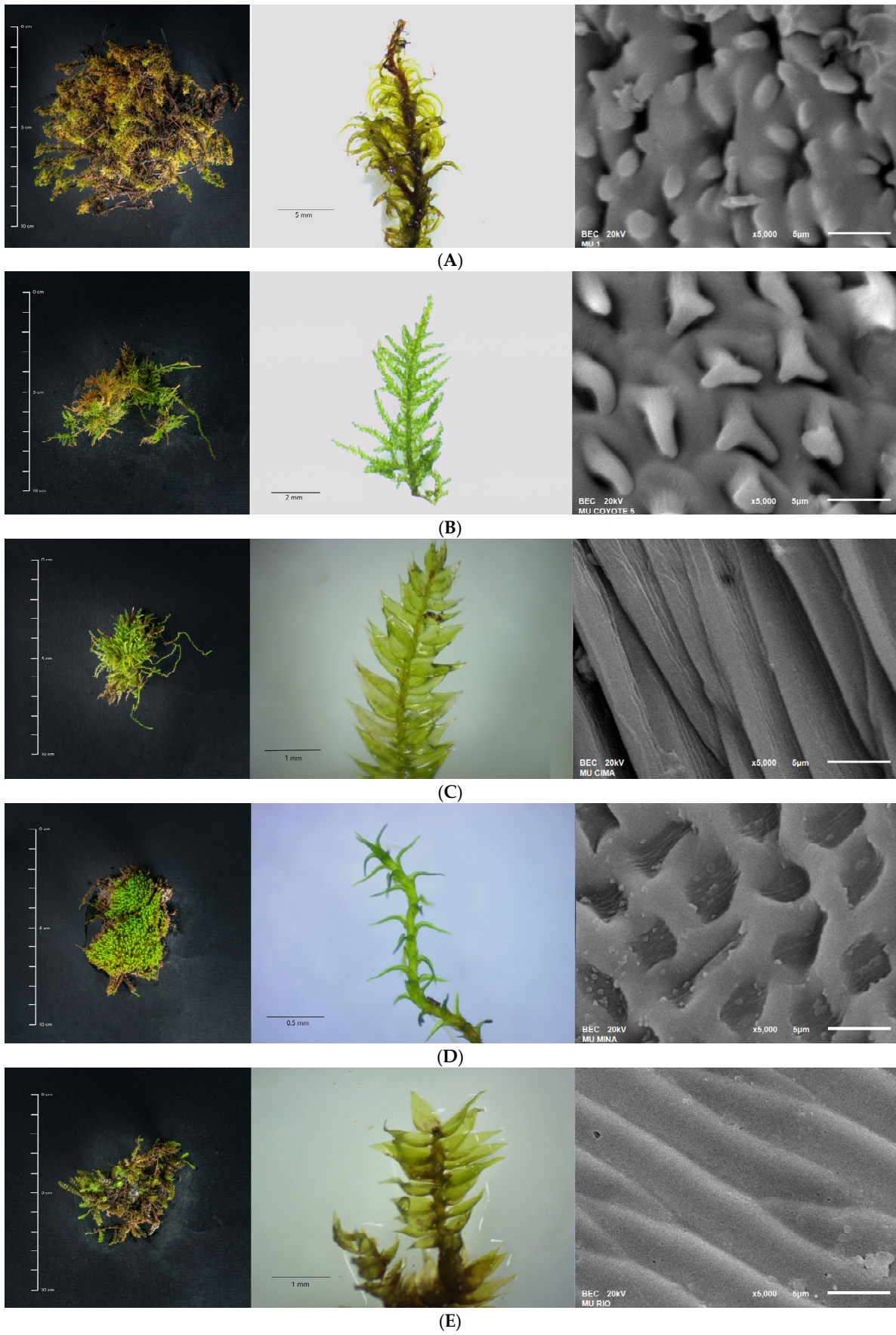

Figure S2: Moss species

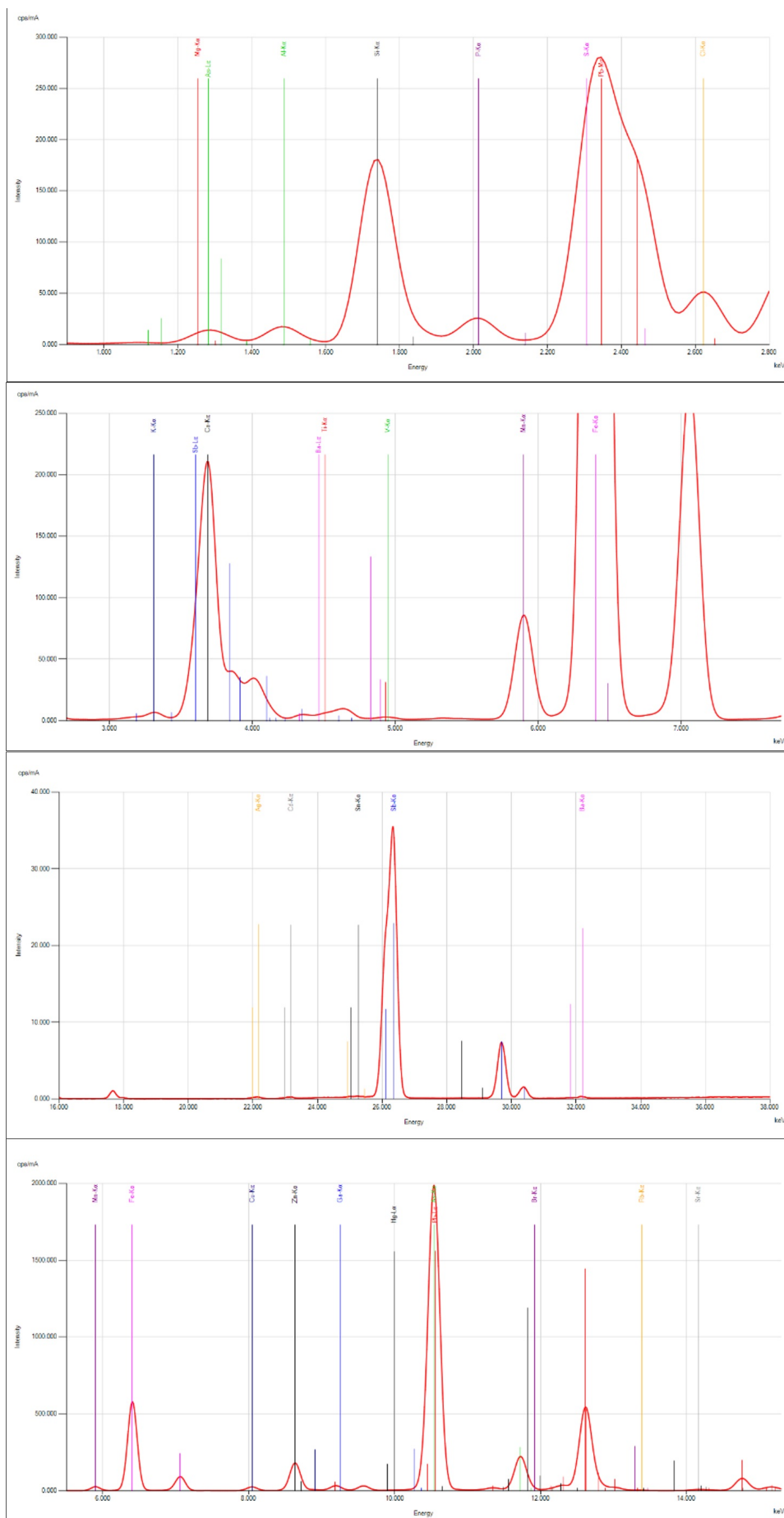

Figure S3: EDXRF analysis

Supplement: Supplementary file 1 [file plants-14-02657-s001.zip › figures.pdf]
